# Hsa-circ-0007292 promotes the osteogenic differentiation of posterior longitudinal ligament cells via regulating SATB2 by sponging miR-508-3p

**DOI:** 10.18632/aging.203381

**Published:** 2021-08-23

**Authors:** Anlong Jiang, Nanxiang Wang, Xinxing Yan, Yunheng Jiang, Chengchao Song, Hui Chi, Guanghua Chen, Feng Wu, Ye Ji, Jinglong Yan

**Affiliations:** 1 Department of Orthopedics, The Second Affiliated Hospital of Harbin Medical University, Harbin, China; 2 The Key Laboratory of Myocardial Ischemia, Harbin Medical University, Ministry of Education, Harbin, China

**Keywords:** ossification, posterior longitudinal ligament, circRNA, osteogenic differentiation, ceRNA

## Abstract

Ossification of the posterior longitudinal ligament (OPLL) is a disorder with multiple pathogenic mechanisms and leads to different degrees of neurological symptoms. Recent studies have revealed that non-coding RNA (ncRNA), including long non-coding RNAs (lncRNAs) and microRNAs (miRNAs), could influence the development of OPLL. Nevertheless, the molecular mechanisms linking circular RNAs (circRNAs) and the progression of OPLL is still unknown. The current research explored the expression profiles of OPLL-related circRNAs by microarray analysis, and applied qRT-PCR to validate the results. Subsequently, we confirmed the upregulation of hsa_circ_0007292 in OPLL cells by qRT-PCR and validated the circular characteristic of hsa_circ_0007292 by Sanger sequencing. Fluorescence *in situ* hybridization (FISH) unveiled that hsa_circ_0007292 was predominantly located in the cytoplasm. Functionally, gain-of-function and loss-of-function experiments showed that hsa_circ_0007292 promoted the osteogenic differentiation of OPLL cells. Mechanistically, the interaction of hsa_circ_0007292 and miR-508-3p was predicted and validated by bioinformatics analysis, dual-luciferase reporter assays, and Ago2 RNA immunoprecipitation (RIP). Similarly, we validated the correlation between miR-508-3p and SATB2. Furthermore, rescue experiments were performed to prove that hsa_circ_0007292 acted as a sponge for miR-508-3p, and SATB2 was revealed to be the target gene of miR-508-3p. In conclusion, our research shows that hsa_circ_0007292 regulates OPLL progression by the miR-508-3p/SATB2 pathway. Our results indicate that hsa_circ_0007292 can be used as a promising therapeutic target for patients with OPLL.

## INTRODUCTION

Ossification of the posterior longitudinal ligament (OPLL) is a common orthopedic disease, which is characterized by the progressive heterotopic ossification of the posterior longitudinal ligament (PLL) and frequently involves the cervical spinal cord [1]. OPLL can cause neurological pain and dysfunction by narrowing the vertebral canal and compressing the nerve roots or spinal cord [2]. Once the onset of myelopathic symptoms has started, neurologic function gradually deteriorates, at which point patients require decompressive surgery [3]. However, the symptoms may reoccur postoperatively because of the gradual progression of ossification over time. There is no effective way to prevent the formation and the development of OPLL [4]. Thus, it is important to find a way to inhibit the progression of ossified ligaments, which could be a better way to treat patients with OPLL.

Due to its high prevalence and incidence [5], OPLL has been extensively investigated worldwide. OPLL is regarded as a multi-pathogenesis disease, as nongenetic and genetic factors participate in its progression [6]. The genetic factors contributing to OPLL include OPLL susceptibility genes and signaling pathways [7]. Moreover, accumulating evidence has indicated that long non-coding RNAs (lncRNAs) and microRNAs (miRNAs), two classes of non-coding RNAs (ncRNAs) that participate in posttranscriptional regulation in many diseases [8, 9], are also contributed to the progression of OPLL [10, 11].

Circular RNAs (circRNAs) are another unique class of ncRNAs that were first identified in the 1970s [12] and are produced by the covalent linkage of linear RNA via noncanonical splicing called backsplicing [13]. Due to their closed-loop structures, circRNAs are more conserved and stable than lncRNAs and miRNAs [14]. The development of sequencing technologies and bioinformatics tools has resulted in an ever-increasing understanding of the diseases and biological functions related to circRNAs. The primary biological functions of circRNAs involves transcriptional regulation, miRNA sponges, interaction with proteins, and even translation into proteins [15]. Previous studies unveiled that circRNAs not only play important roles in the progression, migration, and metastasis of many cancers [16, 17] but also participate in various orthopedic diseases. For instance, circVMA21 can alleviate intervertebral disc degeneration [18], hsa_circ_0026827 can promote osteoblast differentiation, which provides novel therapeutic targets for osteoporosis treatment [19]. In particular, a previous study identified the differentially expressed circRNAs in ossification of ligamentum flavum (OLF) [20], which is a spinal disorder remarkably similar to OPLL [21].

However, as far as we know, few studies have explored the mechanism of circRNAs in regulating the development of OPLL. Therefore, the principal aim of our research was to unveil the important role of circRNAs in regulating the development of OPLL. We applied circRNA microarray assays, and first reported a novel OPLL-related circRNA, hsa_circ_0007292, which showed a higher expression level in OPLL tissues than normal tissues and participated in the pathogenesis and development of OPLL by targeting the miR-508-3p/SATB2 pathway. Our results will provide a new insight into the regulatory mechanisms of hsa_circ_0007292 in the development of OPLL.

## RESULTS

### Differentially expressed circRNA profiles in OPLL

**Figure 1:**
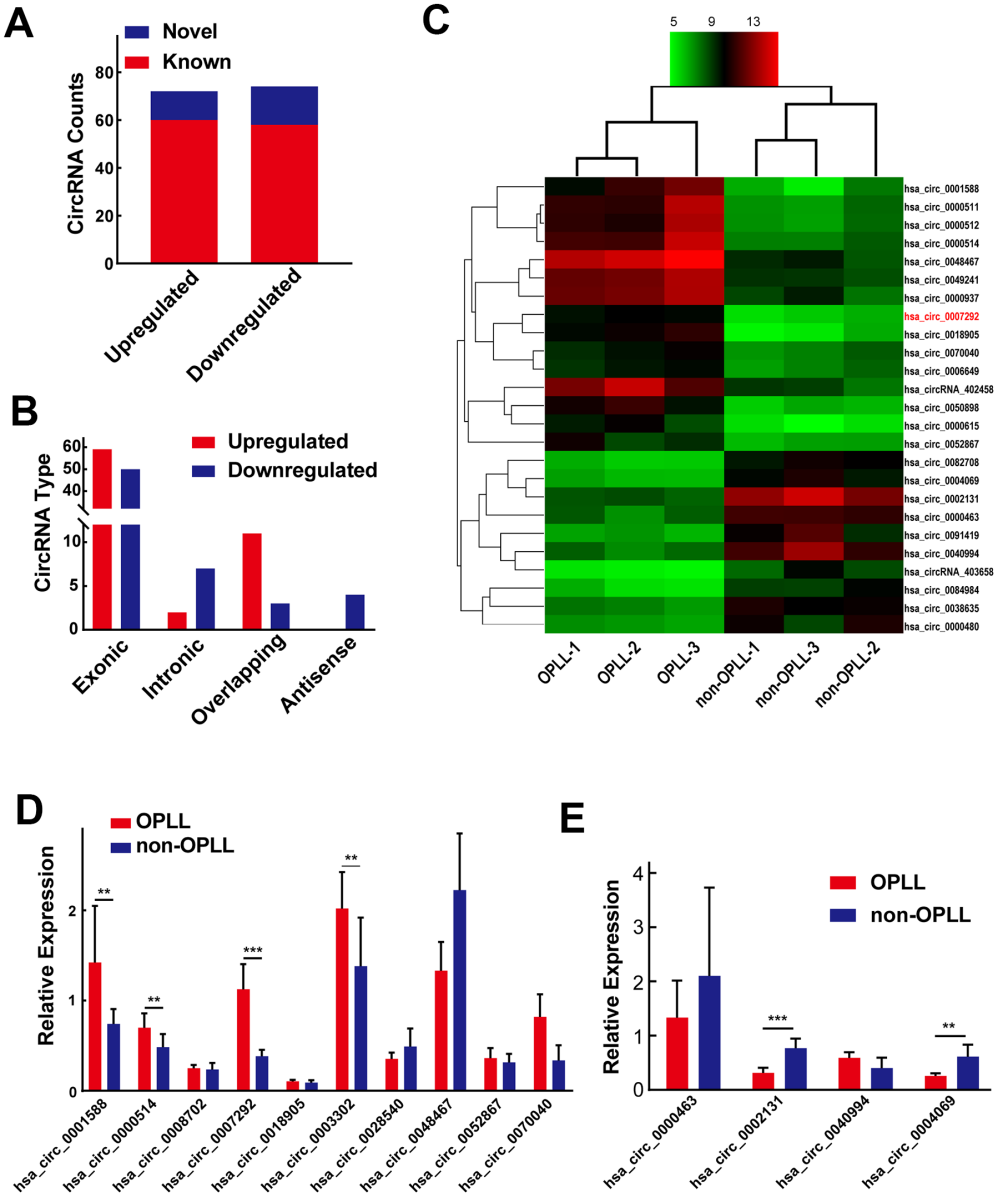
**Differentially expressed circRNA profiles in OPLL.** (**A**) Of all the 146 identified circRNAs, 28 of them were confirmed as novel circRNAs; 118 of them were previously reported in the database of circBase. (**B**) The 146 dysregulated circRNAs were classified into four different groups based on their origins. (**C**) The heatmap shows the top 15 upregulated and top 10 downregulated circRNAs between three OPLL and three non-OPLL tissue samples. (**D**, **E**) The expression of top 10 upregulated and top 4 downregulated circRNAs was measured by qRT-PCR (n=10). Data are expressed as the mean ± SD. ***p < 0.001; **p < 0.01.

To explore OPLL-related circRNAs, the expression profiles of three OPLL and three non-OPLL tissues samples were analyzed by using a circRNA microarray. We recognized 72 significantly upregulated circRNAs and 74 downregulated circRNAs in OPLL tissue samples compared to non-OPLL tissue samples, according to our predefined thresholds of fold change >2.0 and p value <0.05. The heat map and the volcano graph indicated differentially expressed circRNAs between the OPLL and non-OPLL groups. (Supplementary Figure 1A, 1B). We subsequently classified the 146 dysregulated circRNAs based on the datebase of circBase, 118 circRNAs (80.82%) were reported in other previous studies and the other 28 (19.18%) were novel (Figure 1A). We divided the 146 identified circRNAs into four different categories based on their origin. Exonic CircRNAs originated from exon regions accounted for 74.66% (109/146), intronic circRNAs consisting of intron lariats accounted for 7.16% (9/146) of the circRNAs, sense overlapping circRNAs accounted for 8.22% (12/146) of the circRNAs, and antisense circRNAs accounted for 2.74% (4/146) of the circRNAs (Figure 1B). Subsequently, we performed hierarchical clustering analysis to illustrate the top 15 upregulated and top 10 downregulated circRNAs among the circRNAs assessed (Figure 1C). The expression of 10 upregulated and 4 downregulated circRNAs listed in above hierarchical clustering analysis were confirmed by qRT-PCR using 10 OPLL and 10 non-OPLL tissue samples. Hsa_circ_0001588, hsa_circ_0000514, hsa_circ_0007292, hsa_circ_0003302, hsa_circ_0070040, hsa_circ_0002131, and hsa_circ_0004069 exhibited consistent expression levels with the results of microarray analyses (Figure 1D, 1E).

### Identification of the loop structure and expression levels of hsa_circ_0007292

**Figure 2:**
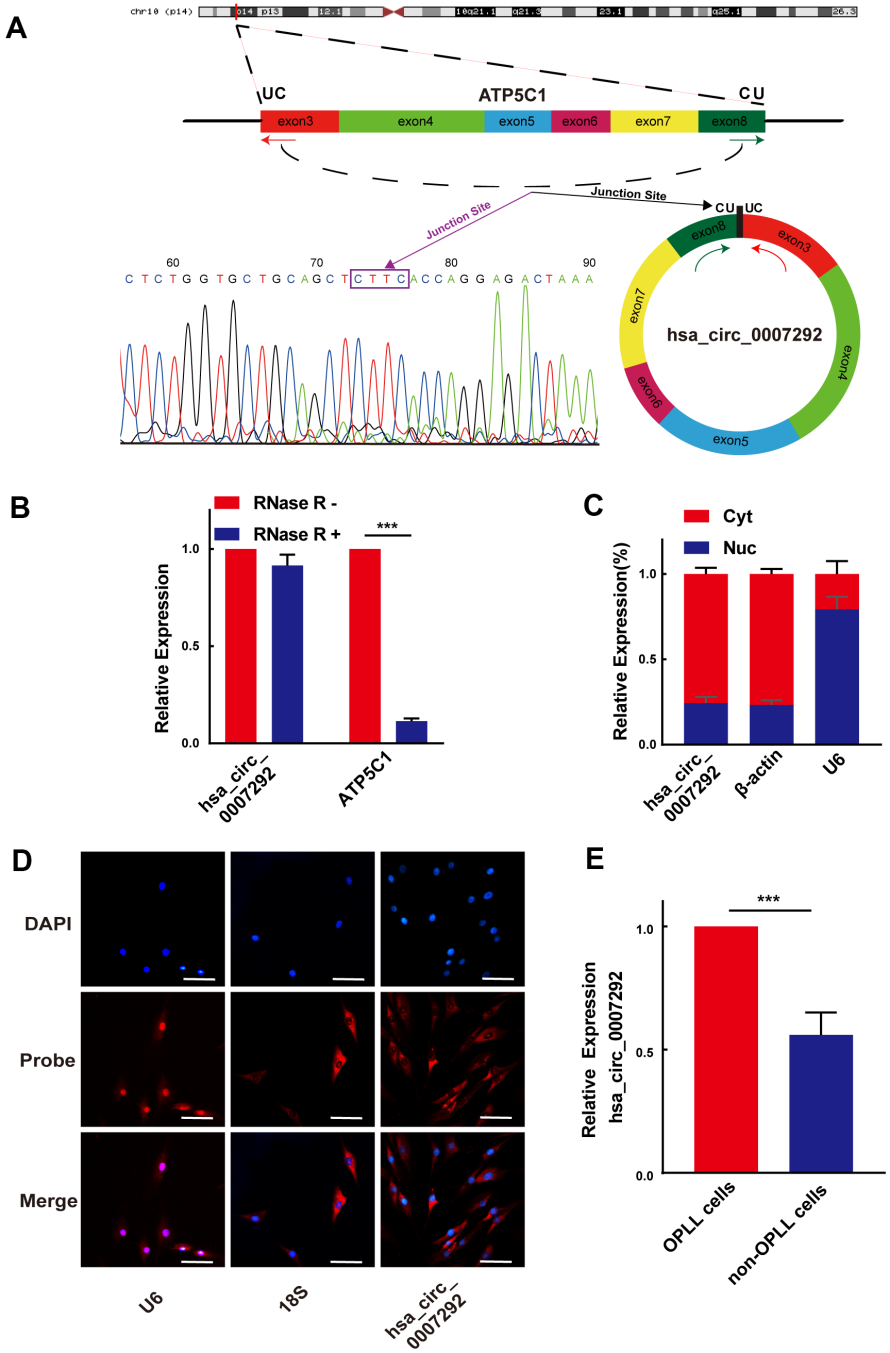
**Identification of the loop structure and expression levels of hsa_circ_0007292.** (**A**) Schematic showing that hsa_circ_0007292 is a circular structure formed from exons 3-8 of ATP5C1 mRNA; the backsplicing site of hsa_circ_0007292 was confirmed by Sanger sequencing. (**B**) Hsa_circ_0007292 could resist the digestion of RNase R in OPLL cells according to the results of qRT-PCR (n=3). (**C**) qRT-PCR experiment of the nuclear (Nuc) and cytoplasmic (Cyt) RNAs revealed that hsa_circ_0007292 was predominantly localized to the cytoplasm. U6 and β-actin were used as the internal control for nuclear RNA and cytoplasmic RNA, respectively (n=3). (**D**) FISH assays indicated that hsa_circ_0007292 was mainly localized to the cytoplasm. The probes of hsa_circ_0007292, U6, and 18S were marked with Cy3, and the nuclei were stained with DAPI. Scale bar, 100 mm. (**E**) Hsa_circ_0007292 was showed a higher expression in OPLL cells (n=8) than in non-OPLL cells detected by qRT-PCR(n=8). All experiments were performed at least three times. Data are expressed as the mean ± SD. ***p < 0.001.

Because hsa_circ_0007292 showed the most significantly upregulation, we choose hsa_circ_0007292 for further research. The detailed information of hsa_circ_0007292 was obtained from circBase, which is a professional bioinformatic database focused on circRNAs. Hsa_circ_0007292 originates from the ATP synthase F1 subunit gamma (ATP5C1) gene located on chr10:7839009-7844817, which is generated by backsplicing of exons 3–8 and has a length of 799bp. The hsa_circ_0007292 backsplicing junction point was verified by Sanger sequencing of the PCR products (Figure 2A). As described in previous studies [22], the primary cells from the PLL of OPLL patients (OPLL cells) and non-OPLL patients (non-OPLL cells) were isolated and cultured for further detecting the effect and mechanism of hsa_circ_0007292 in OPLL. The primary OPLL cells and non-OPLL cells displayed fibroblast-like spindle-shaped cells (Supplementary Figure 2A), and the immunofluorescence staining of vimentin was positive (Supplementary Figure 2B). To further assess the circular characteristics of hsa_circ_0007292, we extracted the total RNA from OPLL cells and divided them into two groups for qRT-PCR: one group was treated with 3′–5′ exoribonuclease (RNase R), and the untreated group was used as a control. The corresponding linear mRNA ATP5C1 could not resistant to RNase R digestion compared to the control group, while hsa_circ_0007292 expression level was not statistically changed (Figure 2B). Subsequently, we validated the relative expression levels of hsa_circ_0007292 in the cytoplasm and nucleus by qRT-PCR to detect its subcellular distribution. The results confirmed that hsa_circ_0007292 was predominantly expressed in the cytoplasm of OPLL cells (Figure 2C). In addition, we validated that hsa_circ_0007292 was predominately localized to the cytoplasm by fluorescence *in situ* hybridization (FISH) (Figure 2D). Moreover, the expression levels of hsa_circ_0007292 was higher in OPLL cells than that in non-OPLL cells (Figure 2E). In summary, these findings indicate that hsa_circ_0007292 may have potential biological functions in post-transcriptional regulation of the pathogenetic process of OPLL.

### Hsa-circ-0007292 promotes the osteogenic differentiation of PLL cells

**Figure 3:**
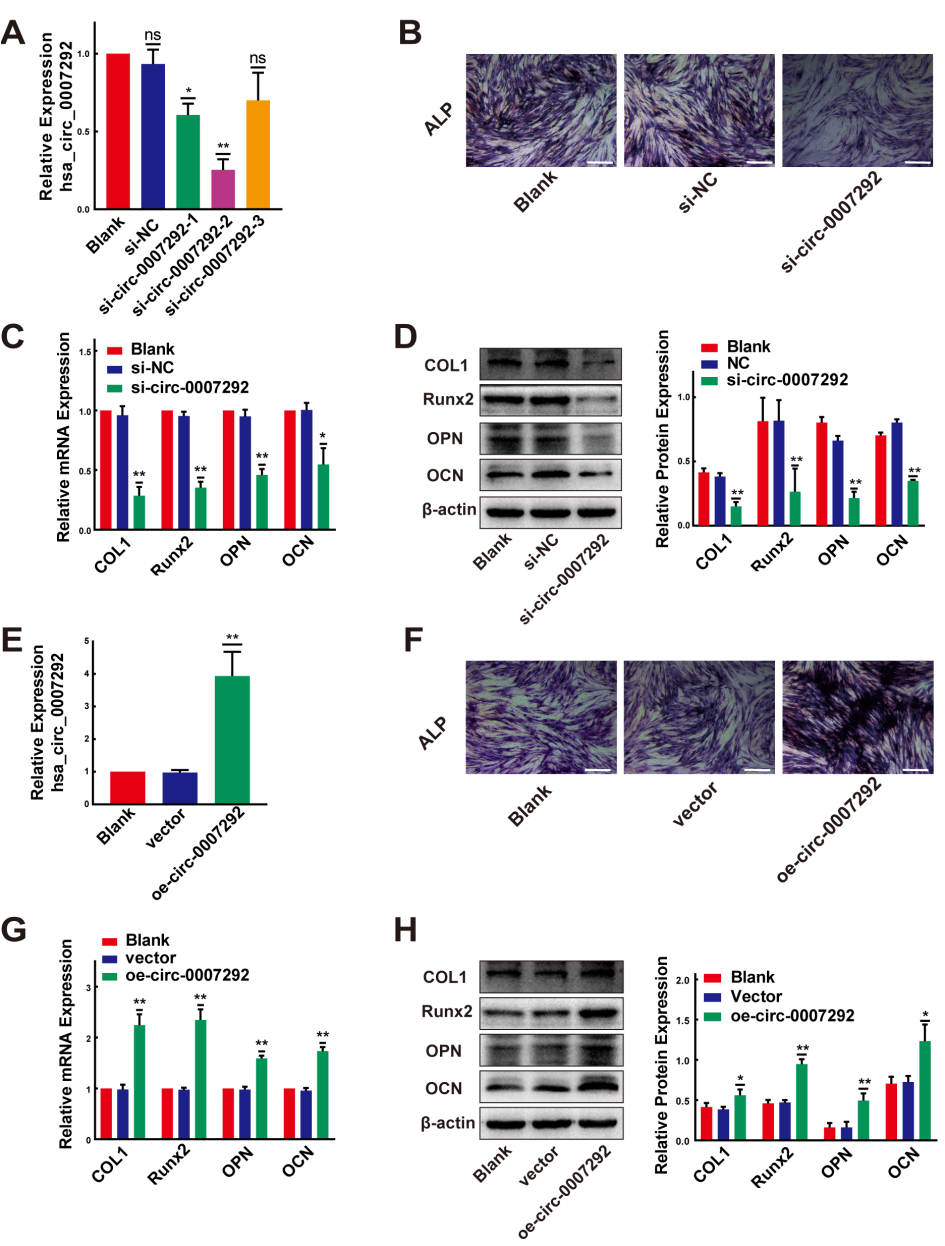
**Hsa-circ-0007292 promotes the osteogenic differentiation of PLL cells.** (**A**) qRT-PCR assays detected the transfection efficiency of siRNAs targeting hsa_circ_0007292 in OPLL cells (n=3). (**B**) The osteogenic properties of OPLL cells transfected with siRNA of hsa_circ_0007292 and negative control were analyzed using alkaline phosphatase staining after osteo-induction for 10 days (scale bars, 100 um). (**C**, **D**) Knockdown of hsa_circ_0007292 inhibited the mRNA and protein expression of osteogenesis-related markers in OPLL cells (n=3). (**E**) Hsa_circ_0007292 was significantly upregulated after transfection of non-OPLLs with the overexpression vector. (**F**) The osteogenic properties of non-OPLL cells transfected with overexpression plasmid and blank vector were analyzed using alkaline phosphatase staining after osteo-induction for 10 days (scale bars, 100 um). (**G**, **H**) Overexpression of hsa_circ_0007292 promoted osteogenesis-related markers expression in non-OPLL cells at both mRNA and protein levels (n=3). All tests were conducted at least three times. Data are exhibited as mean ± SD. ns (not significant, P>0.05), *P < 0.05, **P < 0.01.

We confirmed the osteogenic capabilities of PLL cells before exploring the functions of hsa_circ_0007292. The expression levels of ossification-related markers (COL1, Runx2, OPN, and OCN) were examined by qRT-PCR at diverse time points during osteoinduction. In compliance with previous studies [10, 23, 24], the osteogenic differentiation properties of OPLL cells were better than PLL cells (Supplementary Figure 2C). After detecting the osteogenic properties of PLL cells, three small-interfering RNAs (siRNAs), targeted the junction site of hsa_circ_0007292, were designed to disrupt hsa_circ_0007292 expression. We transfected OPLL cells with the three siRNAs. The expression of hsa_circ_0007292 was notably suppressed by si-circ-0007292-1 and especially decreased by si-circ-0007292-2 but was not affected by hsa_circ_0007292-3 (Figure 3A). However, the linear isoform of hsa_circ_0007292, ATP5C1 mRNA, was not silenced by the three siRNAs (Supplementary Figure 2D). Thus, we selected si-circ-0007292-2 for the subsequent loss-of-function experiments. The ALP activity was suppressed in si-circ-0007292 group compared to the negative control group (Figure 3B). qRT-qPCR and Western Blot detection confirmed that the expression level of ossification-related markers COL1, Runx2, OPN and OCN was significantly inhibited after interference of hsa_circ_0007292 (Figure 3C, 3D). We also constructed an overexpression plasmid for hsa_circ_0007292. The relative expression of hsa_circ_0007292 was meaningfully increased after transfection of non-OPLL cells with the overexpression vector, while the blank vector did not alter expression (Figure 3E); transfection did not affect the levels of linear ATP5C1 mRNA (Supplementary Figure 2E). After treated with hsa_circ_0007292 overexpression plasmid, the ALP activity of PLL cells was promoted significantly (Figure 3F). We next confirmed the osteogenesis-promoting effect of hsa_circ_0007292 by exploring the mRNA and protein levels of the ossification-related markers (COL1, RUNX2, OPN, and OCN) after treated with overexpression vector or blank vector. The qRT-PCR and Western blot results validated that hsa_circ_0007292 overexpression upregulated the expression of ossification-related markers at mRNA and protein levels (Figure 3G, 3H). Altogether, we confirmed that hsa_circ_0007292 could significantly regulate the osteogenic differentiation of PLL cells *in vitro*.

### Hsa_circ_0007292 serves as a sponge for hsa-miR-508-3p

**Figure 4:**
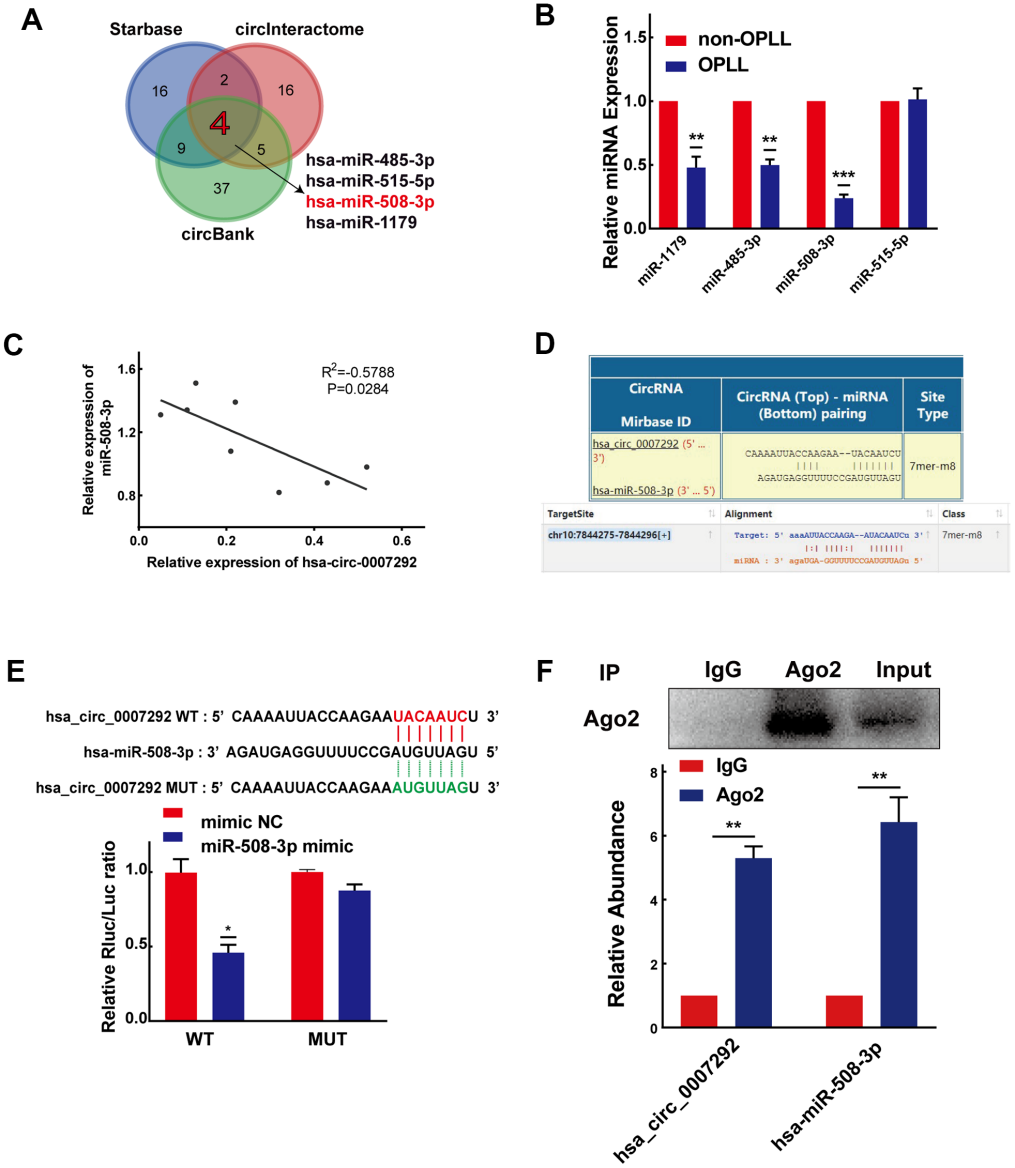
**Hsa_circ_0007292 serves as a sponge for miR-508-3p.** (**A**) Schematic exhibiting the overlapping target miRNAs of hsa_circ_0007292 predicted by the circInteractome, StarBase, and circBank databases. (**B**) The relative expression of the four overlapping miRNAs was investigated by qRT-PCR assay in OPLL cells (n=6) and non-OPLL cells (n=6). (**C**) The correlation between relative miR-508-3p expression and relative hsa_circ_0007292 expression in OPLL cells (n=8, R^2^=-0.5788, P=0.0284). (**D**) Schematic of the predicted target site between miR-508-3p and hsa_circ_0007292 from circInteractome and StarBase online databases. (**E**) The activity of hsa_circ_0007292 dual-luciferase reporter was detected in HEK-293T cells cotransfected with miR-508-3p mimic or mimic control(n=3). (**F**) Hsa_circ_0007292 and miR-508-3p simultaneously showed a higher expression in the anti-Ago2 precipitation product(n=3). All experiments were performed at least three times. Data are expressed as the mean ± SD. *P < 0.05, **P < 0.01, ***P<0.001.

Due to hsa_circ_0007292 is stably expressed in the cytoplasm, we primarily focused on the miRNA sponges of hsa_circ_0007292 to explore its regulatory mechanisms in OPLL. The circInteractome, StarBase v3.0, and circBank bioinformatics prediction tools were applied to explore the possible miRNAs with complementary sequences for binding to hsa_circ_0007292. Finally, 4 miRNAs (miR-1179, miR-485-3p, miR-508-3p, and miR-515-5p) were selected by overlapping the prediction results of the three databases (Figure 4A). We subsequently confirmed the relative expression of the 4 miRNAs by qRT-PCR of cells from 6 OPLL samples and 6 non-OPLL samples, which indicated that miR-508-3p exhibited a higher expression level in non-OPLL cells than that in OPLL cells with the most significant statistical difference(p<0.001) (Figure 4B). The expression of miR-508-3p was negatively correlated with that of hsa_circ_0007292 in OPLL cells (Figure 4C). Based on the predicted complementary sequence between hsa_circ_0007292 and miR-508-3p (Figure 4D), we constructed dual-luciferase reporter vectors with hsa_circ_0007292 fragments containing mutant (MUT) or wild-type (WT) binding site sequences of miR-508-3p (Figure 4E). The MUT or WT vector was then cotransfected into HEK293T cells with miR-508-3p mimic or negative control mimic (mimic NC). The dual-luciferase reporter assay validated that the miR-508-3p mimic significantly suppressed the luciferase activity of the WT reporter but not the MUT reporter, indicating that miR-508-3p could bound hsa_circ_0007292 via complementary target sites (Figure 4E). Since Argonaute2 (Ago2) binds to almost all miRNAs, the RIP assay of anti-Ago2 was performed to further explore whether miR-508-3p could bind to hsa_circ_0007292. As expected, both hsa_circ_0007292 and miR-508-3p showed a higher expression level in the anti-Ago2 group than in the control group (Figure 4F), confirming that hsa-miR-508-3p could bind to hsa_circ_0007292 in OPLL cells. In summary, our findings indicated that hsa_circ_0007292 may serve as a miRNA sponge to suppress miR-508-3p in OPLL cells.

### MiR-508-3p suppresses the osteogenic differentiation of PLL cells

**Figure 5:**
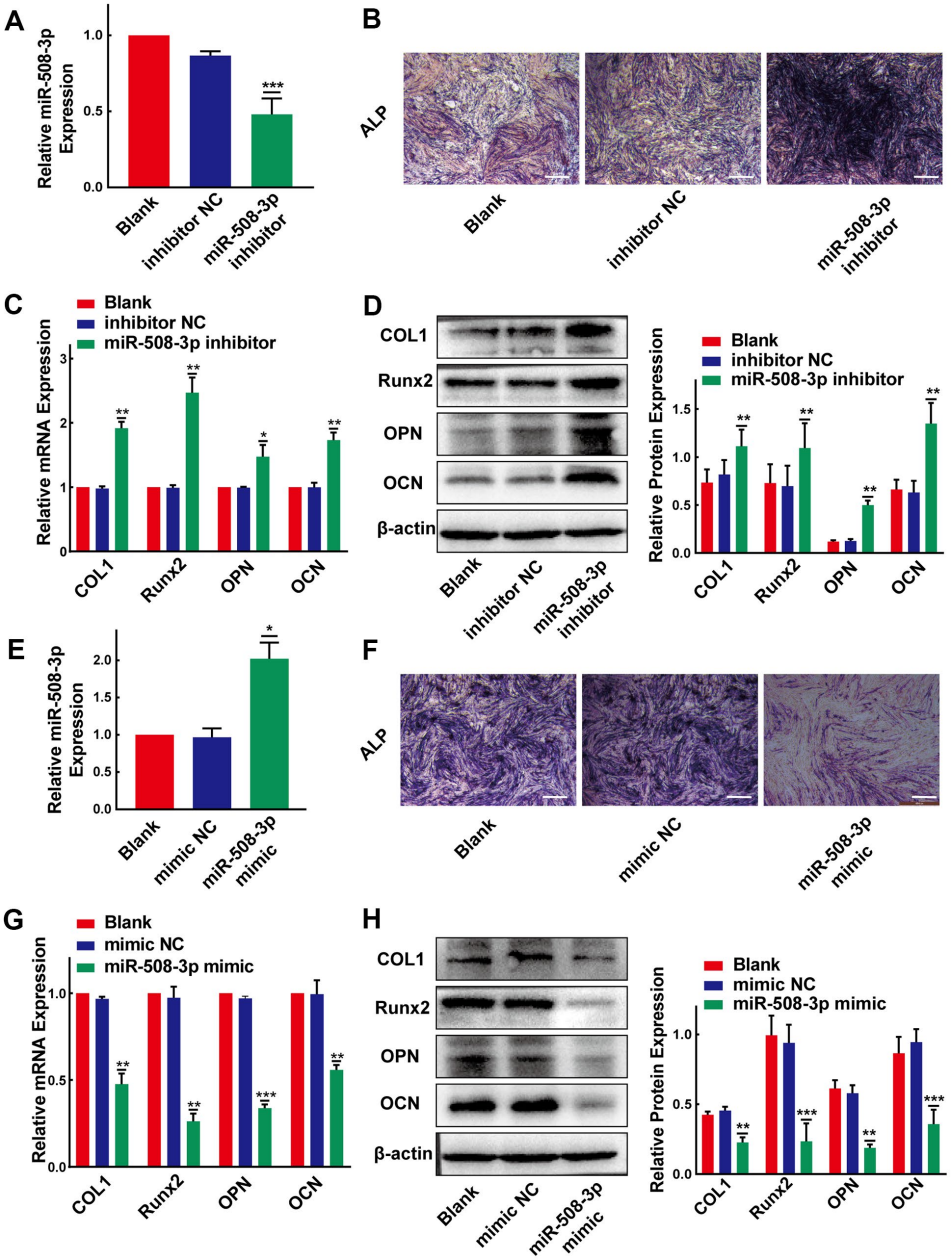
**MiR-508-3p suppresses the osteogenic differentiation of PLL cells.** (**A**) The level of miR-508-3p was significantly suppressed after treatment with the miR-508-3p inhibitor compared to treatment with the negative control(n=3). (**B**) The ALP staining showed that the osteogenic properties of non-OPLL cells were enhanced after treated with miR-508-3p inhibitor. (**C**, **D**) An increased expression of osteogenic differentiation-related markers was detected by qRT-PCR and Western Blot(n=3). (**E**) MiR-508-3p was successfully overexpressed after treated OPLL cells with the miR-508-3p mimic(n=3). (**F**) The ALP activity of OPLL cells was suppressed by miR-508-3p mimic. (**G**, **H**) The expression of osteogenic differentiation-related markers was suppressed at the mRNA and protein levels after transfection with the miR-508-3p mimic versus the negative control(n=3). All tests were conducted at least three times. Data are expressed as the mean ± SD. *P < 0.05, **P < 0.01, ***P<0.001.

Considering that miR-508-3p is the hsa_circ_0007292-targeted miRNA in OPLL cells, we assumed that miR-508-3p might also play significant roles in the process of osteogenic differentiation of PLL cells. To detect the osteogenic effects of miR-508-3p, we successfully silenced and overexpressed miR-508-3p using a miRNA inhibitor and mimic, respectively (Figure 5A, 5E). The ALP activity (Figure 5B) and the mRNA and protein expression level of ossification-related markers (COL1, Runx2, OPN and OCN) were obviously enhanced after miR-508-3p knockdown in non-OPLL cells (Figure 5C, 5D). In contrast, after transfected with the miR-508-3p mimic into OPLL cells, the ALP staining indicated that the ALP activity was suppressed (Figure 5F); the qRT-PCR and Western blot results also showed that the mRNA and protein expression levels of ossification-related markers (COL1, Runx2, OPN and OCN) were obviously reduced during osteogenic differentiation (Figure 5G, 5H). Overall, we confirmed that miR-508-3p could strongly participate in the process of osteogenic differentiation of both OPLL and non-OPLL cells.

### Hsa_circ_0007292 promotes the osteogenic differentiation of PLL cells by sponging miR-508-3p

**Figure 6:**
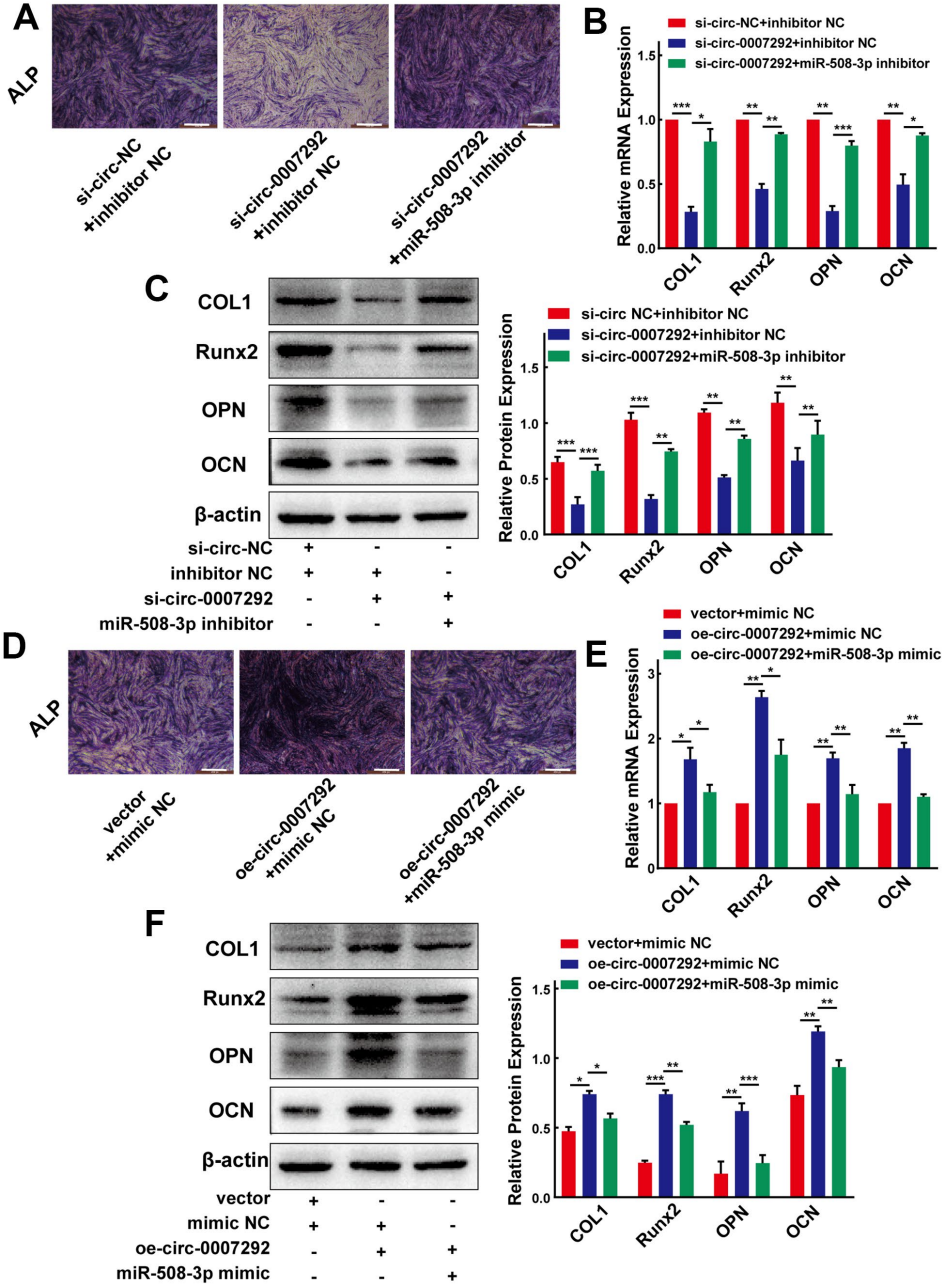
**Hsa_circ_0007292 promotes the osteogenic differentiation of PLL cells by sponging miR-508-3p.** (**A**) The suppressed ALP staining of OPLL cells caused by si-circ-0007292 was rescued by miR-508-3p-inhibitor. (**B**, **C**) The hsa_circ_0007292 knockdown-induced decrease of osteogenic differentiation-related markers was partially restored by miR-508-3p inhibitor at both the mRNA and protein levels(n=3). (**D**) The enhanced ALP staining of non-OPLL cells treated with hsa_circ_0007292 overexpression plasmid was reversed by miR-508-3p mimic. (**E**, **F**) The miR-508-3p mimic rescued the osteogenic effects caused by hsa_circ_0007292 overexpression, as explored by qRT-PCR and Western Blot(n=3). All experiments were conducted at least three times. Data are expressed as the mean ± SD. *P < 0.05, **P < 0.01, ***P<0.001.

To gain insight into whether hsa_circ_0007292 affects the osteogenic differentiation of PLL cells via regulation of miR-508-3p, we further applied the rescue experiments. We cotransfected OPLL cells with miR-508-3p inhibitor and siRNAs targeting hsa_circ_0007292 to examine whether the osteogenic differentiation effect of hsa_circ_0007292 knockdown could be rescued by the miR-508-3p inhibitor. The ALP activity of OPLL cells was inhibited by si-circ-0007292 which could be rescued by miR-508-3p inhibitor (Figure 6A). And the qRT-PCR and Western blot assays validated that the mRNA and protein expression of the osteogenic markers COL1, RUNX2, OPN and OCN significantly reduced after knockdown of hsa_circ_0007292, but the expression of these markers were restored after treatment with the miR-508-3p inhibitor (Figure 6B, 6C). We subsequently performed the miR-508-3p mimic and hsa_circ_0007292 overexpression vector cotransfection experiments to validate the regulatory mechanism between hsa_circ_0007292 and miR-508-3p. The ALP staining experiments revealed that the osteogenic phenotypes of non-OPLL cells were enhanced by overexpression of hsa_circ_0007292, which were reversed by miR-508-3p mimic (Figure 6D). Meanwhile, qRT-PCR and Western Blot experiments indicated that the hsa_circ_0007292 overexpression-mediated promotion of osteogenic markers in non-OPLL cells could be significantly reversed by the miR-508-3p mimic (Figure 6E, 6F). These results suggest that hsa_circ_0007292 might fulfil its functions as a miRNA sponge by targeting miR-508-3p in OPLL cells and that miR-508-3p is a crucial downstream targeting gene for hsa_circ_0007292 that can also play an antagonizing role against hsa_circ_0007292 in PLL cells.

### MiR-508-3p represses SATB2 expression by targeting the 3’-UTR of SATB2

**Figure 7:**
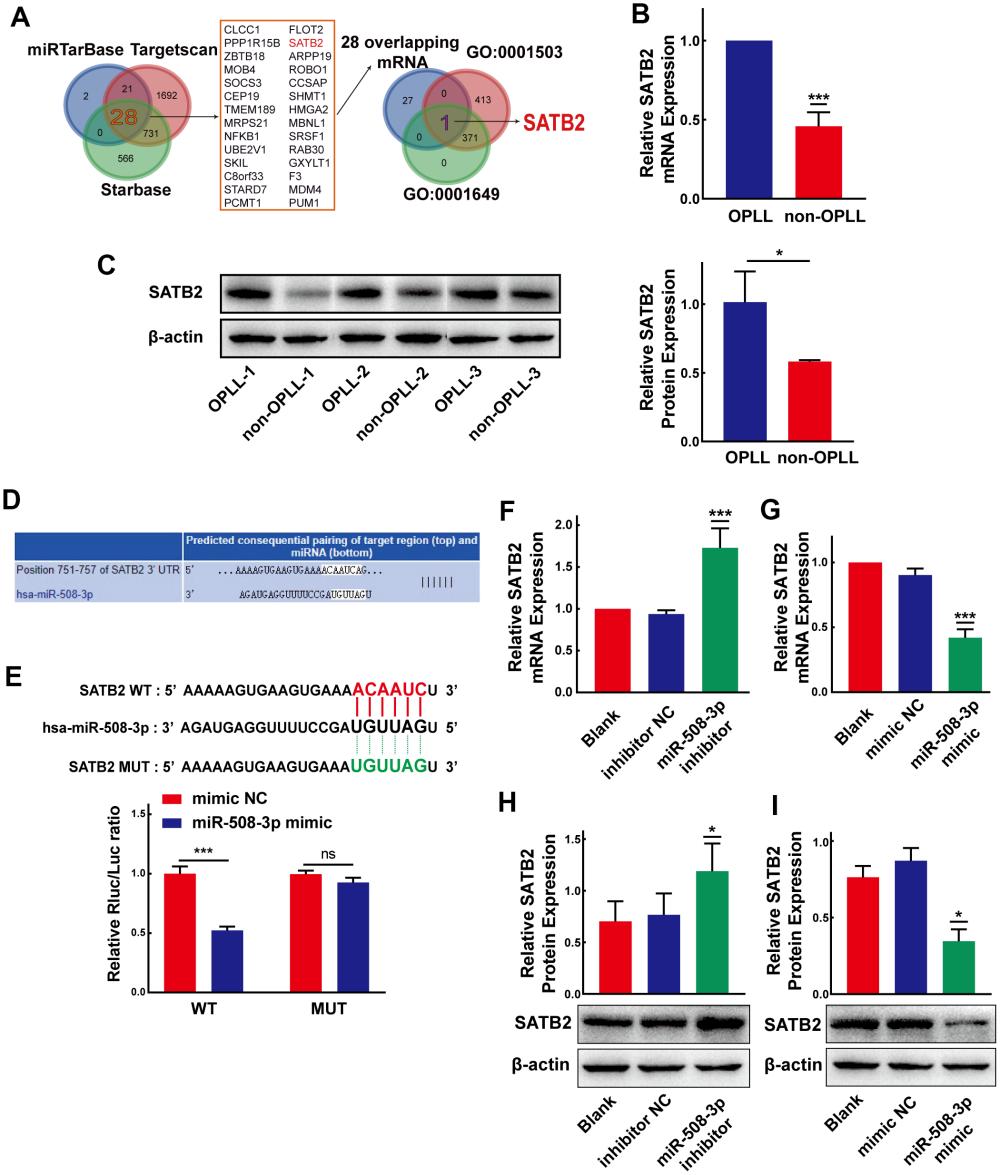
**MiR-508-3p represses SATB2 expression by targeting the 3’-UTR of SATB2.** (**A**) Schematic exhibiting the 28 overlapping target mRNA of miR-508-3p predicted by the miRTarbase, Targetscan, and StarBase databases. The 28 mRNAs further overlapped with those related to the Go terms GO:0001503 and GO:0001649. (**B**, **C**) The expression levels of SATB2 in OPLL cells and non-OPLL cells were detected by qRT-PCR (n=8) and Western Blot (performed 3 times in 3 pairs of the samples) assays. (**D**) Schematic of the predicted target site between miR-508-3p and the 3’-UTR of SATB2 from the Targetscan database. (**E**) The luciferase reporter system assays showed that compared with negative control, the miR-508-3p mimic obviously suppressed the luciferase activity of the WT-SATB2 luciferase reporter vector, while the luciferase activity of MUT-SATB2 was not affected by miR-508-3p mimic(n=3). (**F**, **G**) qRT-PCR assays indicated that the expression level of SATB2 was significantly enhanced or suppressed by the miR-508-3p inhibitor or mimic(n=3). (**H**, **I**) MiR-508-3p inhibitor or mimic could promote or suppress SATB2 protein expression, respectively(n=3). All tests were conducted at least three times. Data are expressed as the mean ± SD. *P < 0.05, **P < 0.01, ***P<0.001.

Considering that miRNAs could target 3’-untranslated region (3’-UTR) of mRNAs by base pairing and play significant roles in posttranscriptional regulation of gene expression, we predicted the target mRNAs of miR-508-3p using three online bioinformatic tools: miRTarBase, TargetScan and StarBase v3.0. We identified 28 targets of miR-508-3p by the overlapped prediction results of the three databases. Among the 28 genes, we screened those related to the Gene Ontology (GO) terms “ossification (GO:0001503)” and “osteoblast differentiation (GO:0001649)” to further screen for potential targets of miR-508-3p. As a result, we found that SATB homeobox 2 (SATB2), a multifunctional regulator of the development of osteoblasts, may be a potential target gene of miR-508-3p (Figure 7A). Based on the qRT-PCR and Western Blot experiments, we confirmed that both the mRNA and protein expression levels of SATB2 were higher in OPLL cells than that in non-OPLL cells (Figure 7B, 7C). To validate these results, the dual-luciferase reporter vector with the WT or MUT SATB2 3’-UTR was constructed, which possessing the putative miR-508-3p target site predicted by the TargetScan database (Figure 7D). The results showed that compared to the mimic control, the miR-508-3p mimic significantly decreased the luciferase activity of the reporter comprising the WT 3’-UTR of SATB2, but the luciferase activity detected in the MUT 3’-UTR of SATB2 group showed no significant changes (Figure 7E). In addition, qRT-PCR and Western blot experiments were applied to validate the results of dual-luciferase experiment. The qRT-PCR result revealed that the miR-508-3p inhibitor and mimic remarkably promoted and inhibited SATB2 mRNA expression, respectively (Figure 7F, 7G). Similarly, the SATB2 protein expression level was also increased or decreased after treatment with the miR-508-3p inhibitor or mimic, as detected by Western blot (Figure 7H, 7I). These data indicate that miR-508-3p could directly combine with the 3’-UTR of SATB2 and suppress its translation.

### Hsa_circ_0007292 modulates the osteogenic differentiation of OPLL cells via the miR-508-3p/SATB2 pathway

**Figure 8:**
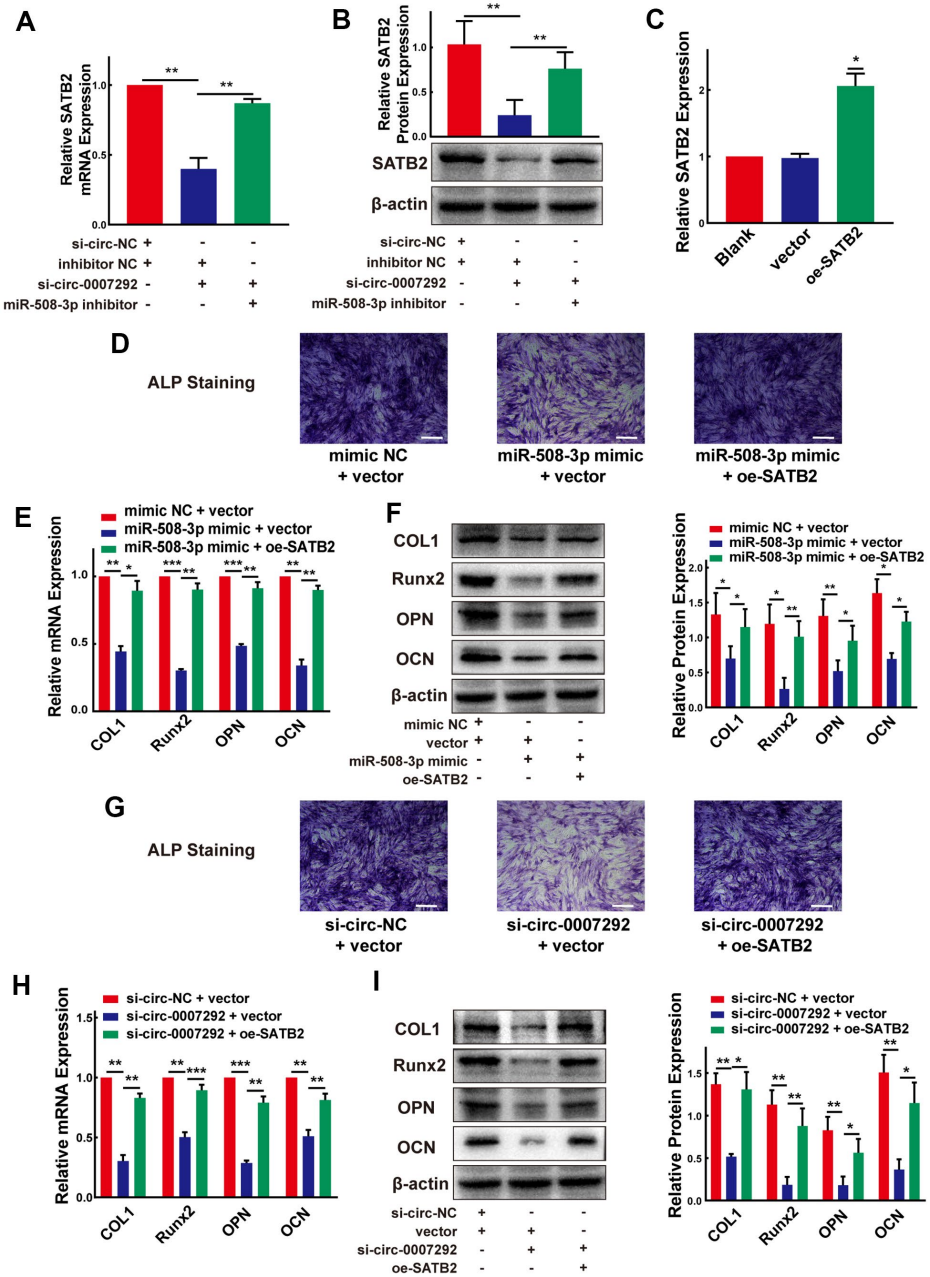
**Hsa_circ_0007292 modulates the osteogenic differentiation of OPLL cells via the miR-508-3p/SATB2 pathway.** (**A**, **B**) The inhibited mRNA and protein expression of SATB2 mediated by hsa_circ_0007292 knockdown was significantly reversed by miR-508-3p inhibitor (n=3). (**C**) SATB2 expression was increased by the SATB2 overexpression vector (n=3). (**D**) The suppressed ALP staining of OPLL cells caused by miR-508-3p mimic was rescued by overexpression of SATB2. (**E**, **F**) The suppression of osteogenic differentiation at the mRNA and protein levels caused by miR-508-3p could be reversed by overexpression of SATB2(n=3). (**G**) The suppressed ALP staining of OPLL cells caused by si-circ-0007292 was rescued by overexpression of SATB2. (**H**, **I**) The overexpression of SATB2 attenuated the hsa_circ_0007292 knockdown-induced inhibitory effects on osteogenesis(n=3). All experiments were conducted at least three times. Data are expressed as the mean ± SD. *P < 0.05, **P < 0.01, ***P<0.001.

After confirming the regulatory mechanisms between hsa_circ_0007292 and miR-508-3p, as well as those between miR-508-3p and SATB2, we further investigated the functions of hsa_circ_0007292 on SATB2 expression when miR-508-3p expression was altered. qRT-PCR and Western blot experiments confirmed that the repressive function of hsa_circ_0007292 downregulation on SATB2 expression could be neutralized by the miR-508-3p inhibitor (Figure 8A, 8B). Furthermore, to validate whether SATB2 is the downstream target of hsa_circ_0007292 and miR-508-3p, a SATB2 overexpression plasmid was constructed, and the SATB2 expression level was significantly increased after transfection of OPLL cells with the overexpression vector versus the blank vector (Figure 8C). Subsequently, we conducted two rescue experiments. Firstly, we found that the suppression of ALP staining caused by miR-508-3p mimic could be neutralized by SATB2 overexpression in OPLL cells (Figure 8D); And the expression of SATB2 (Supplementary Figure 3A, 3B) and osteogenesis-related markers (COL1, Runx2, OPN and OCN) (Figure 8E, 8F) suppressed by the miR-508-3p mimic could be rescued by SATB2 overexpression in OPLL cells. On the other hand, the inhibition of ALP staining caused by si-circ-0007292 could be rescued by overexpression of SATB2 (Figure 8G); And the reductions in the mRNA and protein expression levels of SATB2 (Supplementary Figure 3C, 3D) and ossification-related markers (COL1, Runx2, OPN and OCN) (Figure 8H, 8I) caused by hsa_circ_0007292 knockdown could also be abolished by SATB2 overexpression in OPLL cells. Together, our findings revealed that SATB2 is a downstream target gene of the hsa_circ_0007292/miR-508-3p axis and that hsa_circ_0007292 positively regulates osteogenic differentiation by interacting with miR-508-3p and SATB2 in OPLL cells.

## DISCUSSION

**Figure 9:**
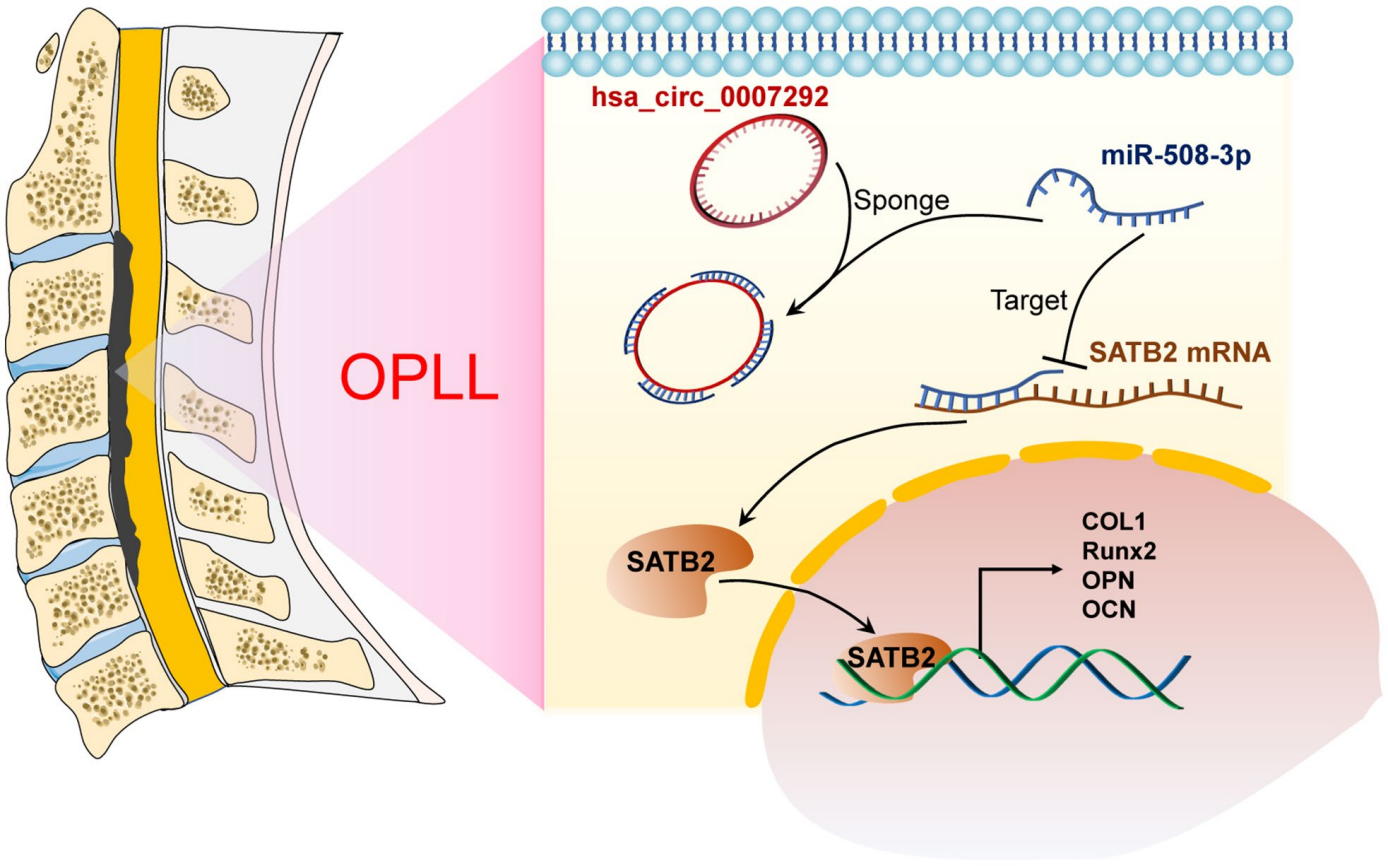
**Schematic of the mechanism of this research.** Schematic exhibiting how hsa_circ_0007292 acting as the miR-508-3p to facilitate the expression of SATB2 and ossification-related genes.

In recent decades, the massive expression levels and regulatory mechanisms of circRNAs have been comprehensively verified in many cancer related diseases [25, 26], and in various orthopedic diseases [27, 28]. However, the function and mechanism of circRNA involved in the development and progression of OPLL are still unknown. In this research, we explored the expression profiles of OPLL-related circRNAs by microarray analysis and mainly paid attention to the function and regulatory patterns of hsa_circ_0007292, which exhibited a higher expression in OPLL tissues and cells. Our results showed that hsa_circ_0007292 could facilitate the progression of OPLL by targeting the miR-508-3p/SATB2 axis (Figure 9).

OPLL is a common orthopedic disorder characterized by the pathophysiological process of calcium deposition at PLL and the ossified PLL can cause spinal cord compression and decreased cervical range of motion [29]. To date, only some lncRNA- and miRNA-related studies have elaborated the functional regulation of OPLL in the field of ncRNAs. For example, miR-182-5p can target the PBX1 gene to promote OPLL development [10]. Some allele combinations of miRNAs might be a genetic pathogenic factor to cervical OPLL [30]. Cai and his colleague [31] unveiled the differential expression of lncRNAs and mRNAs and the potential regulatory mechanisms participated in OPLL, and implied that lncRNAs might be crucial for the pathogenesis of OPLL. Liao’s research team [11] reported that lncRNA XIST might participate in the process of OPLL via the miR-17-5p/BMP2 axis. However, the role of circRNAs involved in OPLL is still unknown.

CircRNAs are an important type of ncRNAs generated by the backsplicing of introns, exons, or intergenic regions and play significant roles in various transcriptional or post-transcriptional regulation due to their high expression in many human tissues and their stable loop structure [32]. Herein, we aimed to explore the potential function and regulatory mechanism of circRNAs involved in OPLL. In current research, we observed that hsa_circ_0007292 exhibited an elevated expression level in OPLL tissues and cells. We also verified the circular characteristics and cytoplasmic localization of hsa_circ_0007292, which indicated that hsa_circ_0007292 might involve in the occurrence and progression of OPLL. Subsequently, loss-of-function assays unveiled that hsa_circ_0007292 knockdown inhibited the osteogenic differentiation of OPLL cells, and gain-of-function assays indicated that hsa_circ_0007292 overexpression facilitated the expression of the osteogenic gene in non-OPLL cells. Our findings confirmed that hsa_circ_0007292 is an OPLL-related circRNA that could positively regulate the osteogenic differentiation of PLL cells.

As accumulating evidences suggest, circRNAs can act as miRNA sponge to regulate various biological processes and gene expression [33, 34]. In the present research, it was verified that hsa_circ_0007292 owned the stable circular structure and predominantly located in cytoplasm. Therefore, we predicted that hsa_circ_0007292 could serve as a sponge of miR-508-3p by bioinformatics tools and validated the interaction between hsa_circ_0007292 and miR-508-3p by luciferase reporter assay and RIP assay. However, only some studies validated that miR-508-3p could exert special biological functions in some cancers [35, 36], the role of miR-508-3p in OPLL remains unknown. In our study, we confirmed that miR-508-3p could suppress the osteogenic differentiation of PLL cells. Moreover, the functional effect of osteogenic differentiation promoted by hsa_circ_0007292 could be rescued by miR-508-3p.

Likewise, we unveiled that SATB2 is a downstream target of miR-508-3p. SATB2 is a transcription factor that can regulate gene expression by modulating chromatin structure [37], which plays significant roles in craniofacial morphogenesis [38], cleft palate formation [39], and many tumor diseases [40]. In particular, SATB2 can regulate osteoblast differentiation by interacting with the transcription factor RUNX2 [41]. In this research, we validated that miR-508-3p could directly interacted with the 3’-UTR of SATB2 by luciferase reporter assay. Further, overexpression of SATB2 rescued the suppression of osteogenic differentiation resulted from knockdown of hsa_circ_0007292 or overexpression of miR-508-3p. The osteogenesis-promoting mechanisms of SATB2 in our study are similar with a previous study [41], which unveiled that SATB2 could promote the osteogenic differentiation of osteoblasts by regulating Runx2 and OCN. Moreover, our findings suggest that hsa_circ_0007292 modulates the expression of SATB2 by sponging miR-508-3p, which is in accordance with the abundant existing circRNA-miRNA-mRNA regulatory mechanisms [42, 43].

In summary, we first reported the upregulation of hsa_circ_0007292 in OPLL tissues and cells. Hsa_circ_0007292 upregulation facilitated the expression of SATB2 through competitively sponging miR-508-3p, thus leading to the disruption of osteogenic differentiation related genes. Our results provide novel understanding into the pathogenesis of OPLL. More importantly, hsa_circ_0007292 might serve as an effective diagnostic target or prognostic biomarker for OPLL, and specific blockade of hsa_circ_0007292 could be a possible prevention or therapy for OPLL in the future. However, we need to design and perform more experiments in the animal model of OPLL to further investigate the significance of hsa_circ_0007292 *in vivo*.

## MATERIALS AND METHODS

### Clinical samples and ethical approval

Our study was approved by the Ethics Committee of the Second Affiliated Hospital of Harbin Medical University (KY2020-073). All subjects involved in this study came from our institute and underwent anterior cervical corpectomy decompression surgery. 18 OPLL and 18 non-OPLL (cervical disc herniation) patients were diagnosed by computerized tomography (CT) and magnetic resonance imaging before surgery. Of all the 18 OPLL patients, 9 of them were localized type, 4 of them were segmental type, 4 of them were mixed type, and 1 of them was continuous type. The detailed information of the research subjects was listed in Supplementary Table 1. All the 36 PLL tissue samples were collected intraoperatively and were used for cell culture or RNA extraction: 8 PLL tissues from the non-ossified area of OPLL patients and 8 PLL tissues from the non-OPLL patients were put into sterilized saline solution for cell culture; 10 PLL tissues from the non-ossified area of OPLL patients and 10 PLL tissues from the non-OPLL patients were frozen in liquid nitrogen as soon as possible (no more than 10 minutes) for subsequent RNA extraction.

### Tissue RNA extraction and circRNA microarray assay

We extracted total RNA from PLL tissues by TRIzol (Invitrogen, USA) based on the commodity’s protocols. Three OPLL and three non-OPLL tissue samples were selected based on the quality and quantity of total RNA. The detailed information of the 6 selected samples were shown in Supplementary Table 2. Next, we prepared the RNA samples for microarray hybridization based on the normative protocols of Arraystar. In brief, linear RNAs needed to be removed to enrich circRNAs by digesting the total RNA with RNase R (Epicenter Technologies, Madison, WI, USA). Subsequently, we amplified the enriched circRNAs into cRNAs, which was labeled and hybridized onto the circRNA microarray (Arraystar Human circRNA Array). Followed by washing the slides, Agilent Scanner G2505C was applied to scan the microarrays. The acquired array images were analyzed by the Agilent Feature Extraction software (version 11.0.1.1). R software limma package was used to process the obtained data.

### Primary cell culture and osteogenic induction

Based on previous studies [44], we isolated and cultured primary PLL cells from PLL tissues. Briefly, the PLL tissues were dissected carefully from a non-ossified site. Then, we minced the tissues into approximately 1-mm^3^ pieces and washed them with sterilized phosphate buffer saline (PBS) (HyClone, South Logan, UT, USA). Afterward, the tissue chips were placed into a T25 culture flask (Corning Life Sciences, New York, NY, USA) without culture medium and placed upside down in the incubator. Approximately 8 hours later, the tissue chips had adhered firmly to the flask. Subsequently, the flask was turned over, and the tissue chips were cultured in Dulbecco’s modified Eagle’s medium (DMEM) (HyClone, South Logan, UT, USA) contained 10% fetal bovine serum (FBS) (ScienCell, Carlsbad, CA, USA). Cells derived from the cultured fragments were digested with trypsin (Gibco, USA) and further cultured at 37° C under a humidified atmosphere of 5% CO2 and 95% air. The PLL cells were stimulated with the osteogenic induction medium for 10 days to analyze their properties of osteogenic differentiation. Based on a previous report [23], we supplemented the complete culture medium with 10^-8^ M dexamethasone, 10 mM β-glycerol phosphate, and 50 μM ascorbic acid (all from Aladdin, China) to acquire the osteogenic induction medium.

### SiRNAs, miRNA oligonucleotides, vector construction and transfection

RiboBio (Guangdong, China) designed and synthesized the siRNAs targeting hsa_circ_0007292 (si-circ-0007292-1: CTCTTCACCAGGAGACTAA; si-circ-0007292-2: TGGTGCTGCAGCTCTTCAC; si-circ-0007292-3: GCAGCTCTTCACCAGGAGA), and miR-508-3p inhibitor and mimic and the respective negative control. We transfected them into PLL cells with the riboFECT CP Transfection Kit (RiboBio, Guangdong, China). The overexpression plasmid for hsa_circ_0007292 and SATB2 were acquired from GeneChem (Shanghai, China). The full-length sequences of hsa_circ_0007292 was inserted into the GV486 vector (GeneChem, Shanghai, China) to construct the overexpression vector (termed oe-circ-0007292), and the empty vector was used as negative control in current research. Likewise, the overexpression vector of SATB2 (termed oe-SATB2) was constructed by inserting the full-length of SATB2 into the GV146 vector (GeneChem, Shanghai, China). The two overexpression vectors were transfected with lipofectamine 3000 (Invitrogen, Carlsbad, CA, USA) following the manufacturer’s protocol. The transiently transfected oligonucleotides and plasmid could only exist in cells for about three to four days; however, the osteogenic properties will be visualized seven of more days after osteogenic induction. Therefore, for the group treated with osteogenic induction medium, cells were transfected again with the oligonucleotides and(or) plasmid 5 days after the first transfection.

### RNA extraction, and qRT-PCR

We extracted total RNA from PLL cells with TRIzol reagent (Invitrogen, Carlsbad, CA, USA) based on the manufacturer's protocol. For mRNA and circRNA, we used 2μg of total RNA to synthesize the cDNA by using a cDNA synthesis kit (Transcriptor First Strand cDNA Synthesis Kit, Roche Diagnostics, Basel, Switzerland). FastStart Universal SYBR Green Master Kit (Roche Diagnostics, Basel, Switzerland) was applied to perform the quantitative real-time PCR (qRT-PCR) to quantify the expression levels of mRNA or circRNA. Then, β-actin served as internal reference to normalize the expression level by calculating with the 2^-∆∆CT^ method. For miRNA analysis, the reverse transcription was conducted by the tailing method. The cDNAs of miRNAs were synthesized by applying the miRcute Plus miRNA First-Strand cDNA Kit (TIANGEN BIOTECH, Beijing, China). Then, based on the protocols, the miRcute Plus miRNA qPCR kit (TIANGEN BIOTECH, Beijing, China) was applied to complete the qRT-PCR experiments that quantify the expression levels of miRNAs. U6 was used as internal reference to normalize the relative miRNA expression using the 2^-∆∆CT^ method. The sequences of primers applied in the current work are listed in Supplementary Table 3.

### RNase R treatment and RNA extraction from nuclear and cytoplasmic

Before RNase R treated experiment, total RNA samples were divided into two groups. By consulting the manufacturer's protocols, one group was pretreated with RNase R(3 U/μg RNA) (Epicenter Technologies, Madison, WI, USA) at 37° C for 30 min. The rest of each sample was served as a control. Subsequently, qRT-PCR was performed to detect the expression of hsa_circ_0007292 and its corresponding linear mRNA ATP5C1. β-Actin from the control group served as the endogenous control for both groups.

The nuclear and cytoplasmic RNA fractions was isolated by using the PARIS Kit (Invitrogen, Carlsbad, CA, USA). Subsequently, the relative expression of hsa_circ_0007292 was explored by qRT-PCR and β-actin served as the cytoplasmic control while U6 served as the nuclear control.

### Western blot analysis

By using RIPA lysis buffer (Beyotime Biotechnology, Beijing, China), total protein lysates of PLL cells were obtained and centrifuged to discard the cell debris. Subsequently, the concentration of supernatant protein was quantified with a BCA kit (Beyotime Biotechnology, Beijing, China). The 10% sodium dodecyl sulfate-polyacrylamide gel electrophoresis (SDS-PAGE) was applied to separate the protein samples (30 μg), which were subsequently transferred to poly-vinylidene difluoride (PVDF) membrane (Millipore, Billerica, MA, USA). After blocked with 5% nonfat milk for an hour, the PVDF membrane was put into the primary antibodies at 4° C overnight. TBST buffer was used to wash the membranes for three times. Then, the membrane was further incubated into secondary antibody. Following sufficient washing with TBST, an electrochemiluminescence (ECL) system (Tanon, Shanghai, China) with ECL Reagent (GE Healthcare, Amersham Biosciences, UK) was applied to detect the protein bands. All the antibodies used in the current studies are shown in Supplementary Table 4. All Western Blot experiments in this paper were performed at least three times, and one representative figure was shown in the paper.

### Alkaline phosphatase (ALP) staining

The BCIP/NBT Alkaline Phosphatase Color Development Kit (Beyotime Biotechnology, Beijing, China) was used to perform the ALP staining experiments. According to the manufacturer’s instructions, cells were fixed with 4% paraformaldehyde after washed with PBS buffer for three times. Subsequently, cells were stained with the BCIP/NBT solution for 30 minutes. Finally, the stained cells were washed with deionized water for 3 times to stop the ALP staining reaction and observed with an inverted microscope. All ALP staining experiments in this paper were performed at least three times, and one representative figure was shown in the paper.

### FISH

FISH experiments were performed to validate the subcellular location of hsa_circ_0007292 by using the Ribo FISH Kit (RiboBio, Guangzhou, China). Probes for hsa_circ_0007292, 18S (served as the internal reference cytoplasm), and U6 (served as the internal reference of nucleus) were acquired from RiboBio (Guangzhou, China). The cells were seeded into a 24-well plate and cultured to 65%–75% confluency before experiments. Then we permeabilized and fixed the cells with 0.5% Triton X-100 and 4% paraformaldehyde. The samples were next cultured with prehybridization solution, followed by incubation in hybridization buffer with specific Cy3-labeled probes for hsa_circ_0007292, 18S, and U6. After thorough washing in SSC buffer, 4’,6-diamidino-2-phenylindole (DAPI) was used to stain the nuclei. A fluorescence microscope (Olympus, Tokyo, Japan) was applied to acquire the images.

### Dual-luciferase reporter assay

The sequences hsa_circ_0007292 and SATB2 were obtained from online database. The pmiR-RB-REPORT plasmid of hsa_circ_0007292-wt, hsa_circ_0007292-mut, SATB2 3’-UTR-wt, and SATB2 3’-UTR-mut were synthesized by RiboBio (Guangzhou, China). Briefly, we seeded the HEK-293T cells into 24-well plates and incubated the cell to 75% density until transfection. According to the group of experiments, we transfected 50ng plasmids and 50nM miRNA mimics or negative control into the cells. After incubated for 48h, we collected the cells for the detection of luciferase activity. Following the manufacturer’s instructions, we applied a dual-luciferase kit (Promega, USA) to detect the activity of firefly luciferase and Renilla luciferase.

### Anti-Ago2 RNA immunoprecipitation (RIP)

The RIP experiment in this study was conducted with a Magna RIP Kit (Millipore, Billerica, MA, USA) in the light of the manufacturer’s protocols. In brief, after the OPLL cells were lysed with RIP lysis buffer, we incubated the RIP lysate with protein A/G magnetic beads, which were pre-conjugated with anti-Ago2 (ab186733, Abcam) or negative control anti-IgG (Millipore) antibody in RIP immunoprecipitation buffer. After RNA extraction, the expression of coprecipitated hsa_circ_0007292 and miR-508-3p was evaluated by qRT-PCR analysis.

### Bioinformatics analysis

We obtained the detailed information of circRNAs from circBase (http://www.circbase.org). Then we predicted the target miRNAs of hsa_circ_0007292 by circInteractome (https://circinteractome.irp.nia.nih.gov/index.html), StarBase (https://web.archive.org/web/20110222111721/http://starbase.sysu.edu.cn/) and circBank (http://www.circbank.cn/index.html) databases. The target mRNAs of hsa-miR-508-3p were predicted using miRTarbase (http://mirtarbase.cuhk.edu.cn/php/index.php), Targetscan (http://www.targetscan.org/vert_72/) and StarBase (https://web.archive.org/web/20110222111721/http://starbase.sysu.edu.cn/). The GO terms “ossification (GO:0001503)” and “osteoblast differentiation (GO:0001649)” were acquired from the Gene Ontology (http://geneontology.org/) databases.

### Statistical analysis

All experiments were performed at least three times, and the data are expressed as the mean ± standard deviation. Prism 8.0 software was used to analyze the data with Student's t-test or one-way ANOVA. P < 0.05 was considered to indicate a statistically significant difference.

## Supplementary Material

Supplementary Figures

Supplementary Tables
