# Supplementary material for: Hsa-circ-0007292 promotes the osteogenic differentiation of posterior longitudinal ligament cells via regulating SATB2 by sponging miR-508-3p
Source: Aging (Albany NY). 2021 Aug 23;13(16):20192–217. doi: 10.18632/aging.203381 (PMC8436939; doi:10.18632/aging.203381)
Supplement: Supplementary Figures [file aging-13-203381-s001.pdf]

SUPPLEMENTARY FIGURES

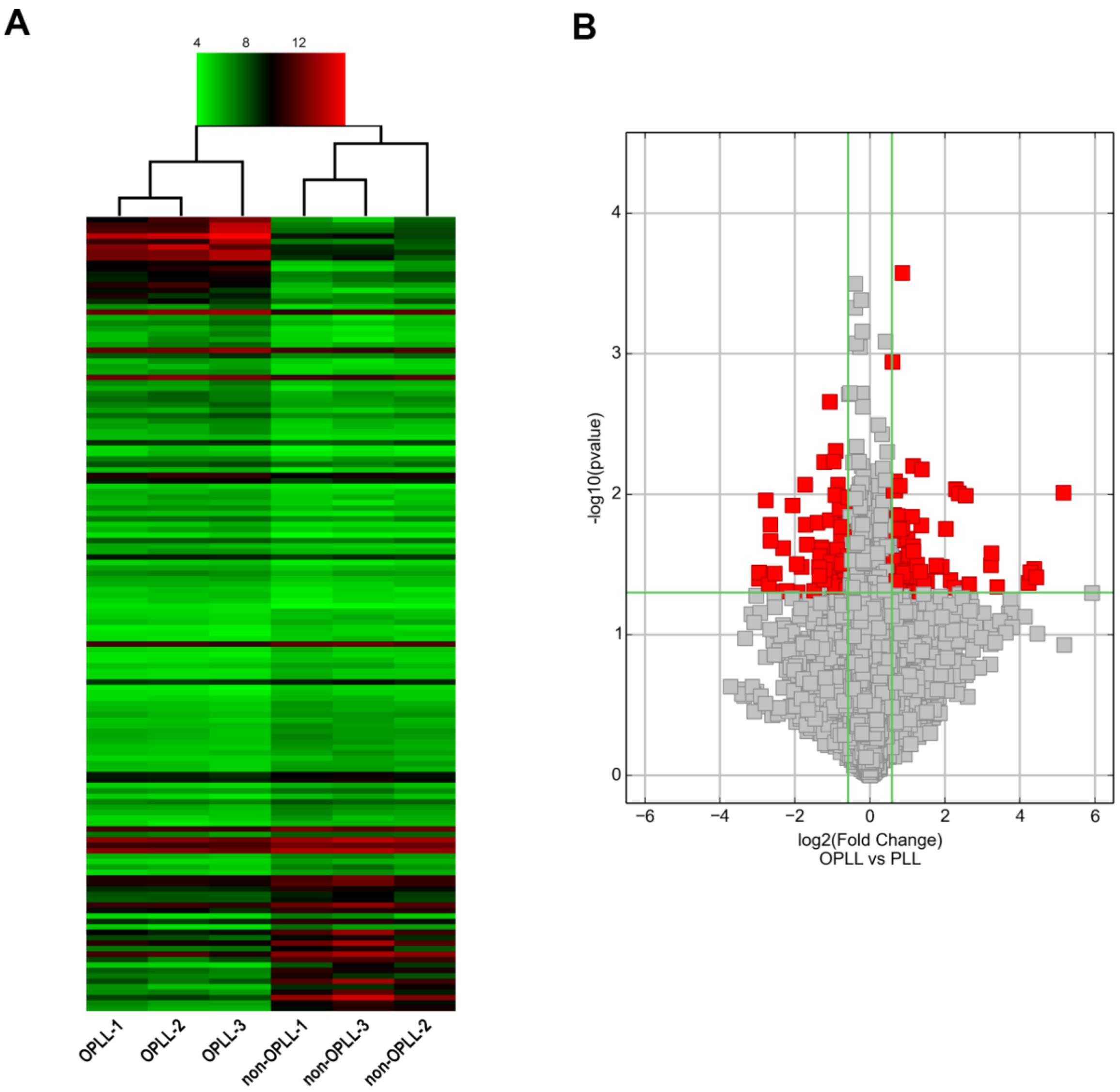

Supplementary Figure 1. Bioinformatic analysis of the results of circRNA microarray analysis. (**A**) Hierarchical clustering of differentially expressed circRNAs in OPLL vs non-OPLL tissue samples. The results of hierarchical clustering show a distinguishable circRNA expression profile among samples. Red: upregulation; green: down-regulation. (**B**) Volcano plots of differentially expressed circRNAs in OPLL vs non-OPLL tissue samples. Volcano plots are useful tools for visualizing differential expression between two different conditions. The red point in the plot represents the statistically significant differentially expressed circRNAs.

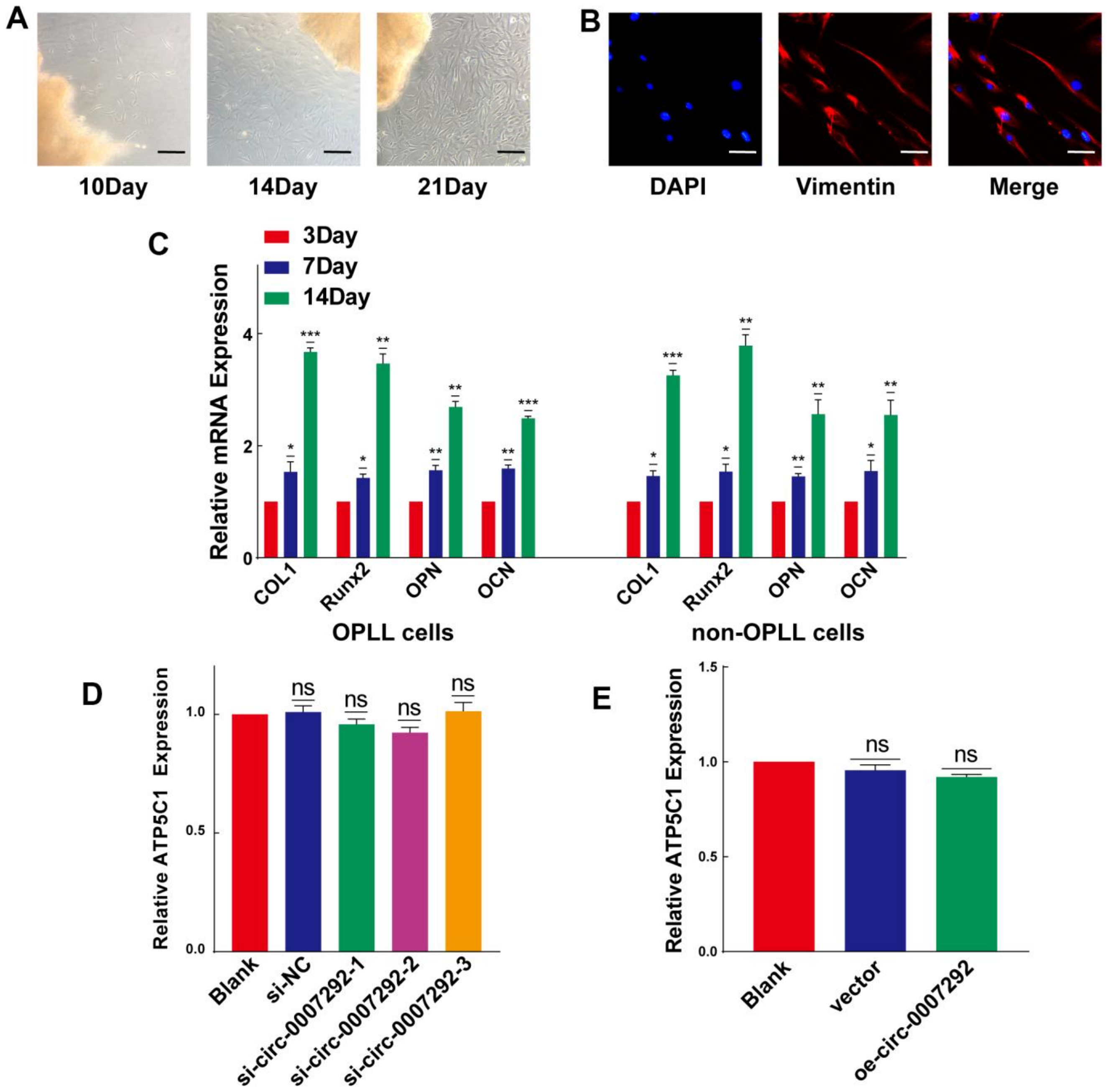

Supplementary Figure 2. Characteristics of PLL ceUs and the effect of siRNA or overexpression vector to ATP5Cl. (**A**) The primary PLL cells were cultured successfully. The fibroblastic cells started to grow out of the tissue chips at the 10th day and could be passaged at the 21th day (scale bars, 100 um). (**B**) Immunofluorescence staining of vimentin(red fluorescence, TRITC labelled) (scale bars, 50 urn). (**C**) Expression of the osteogenetic differentiation related mRNAs in OPLL cells (n=3) and non-OPLL cells (n=3) after osteogenesis induction for 3days, 7days, and 14days. (**D**) The ATP5C I, liner mRNA of hsa_circ_0007292, expression level showed no significantly changed after transfected the three siRNAs of hsa_circ_0007292 (n=3). (**E**) The ATP5C I expression level showed no significantly changed after transfected the overexpression vector of hsa_circ_0007292 (n=3). Data are expressed as the mean ± SD. *p < 0.05, **p < 0.01, ***p < 0.001. NS, not significant (p > 0.05).

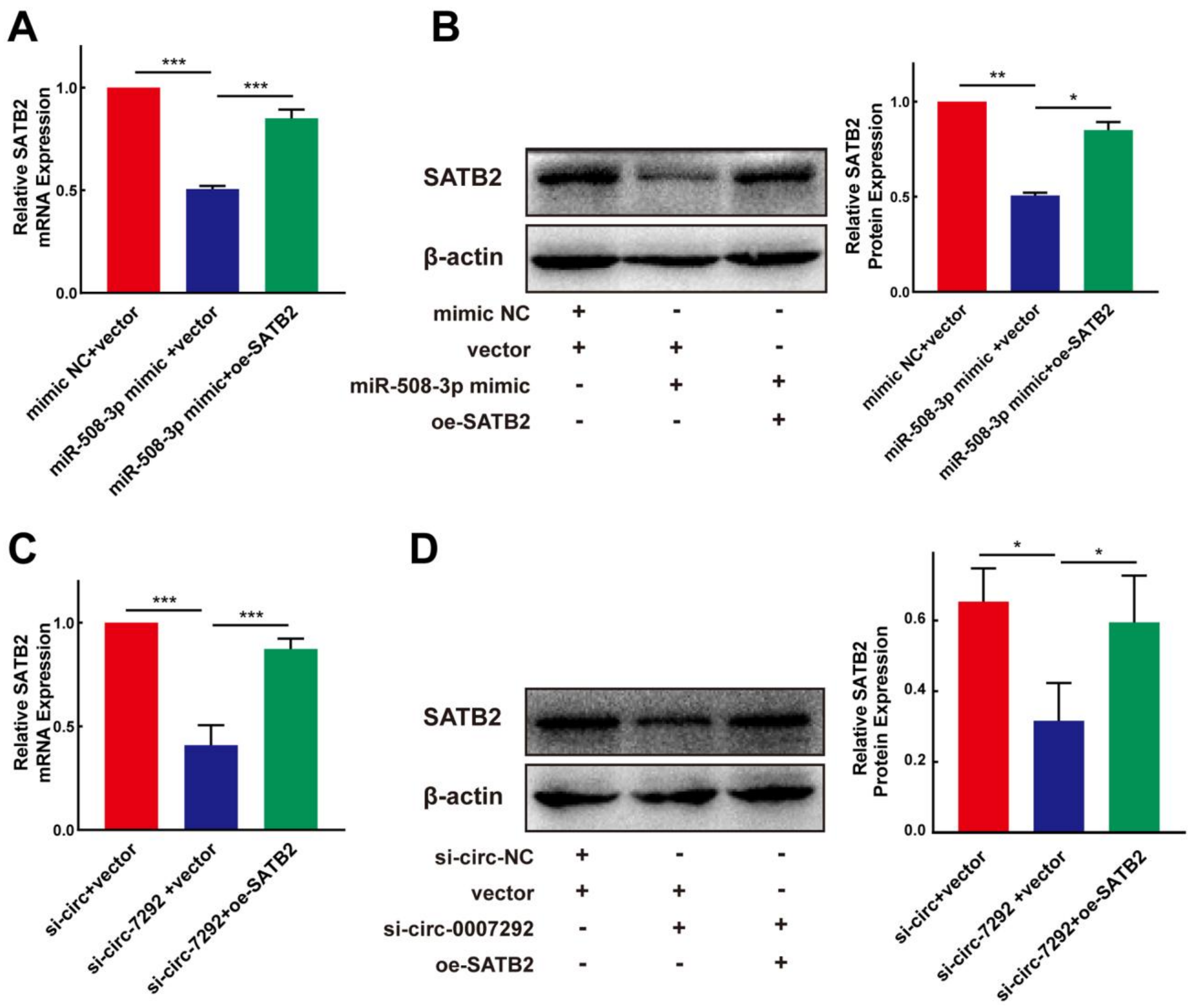

Supplementary Figure 3. The SATB2 expression level after co-transfection of SATB2 overexpression vector and miR-508-3p mimic (or si-circ-7292). (**A**, **B**) The suppression of SATB2 at the mRNA and protein levels caused by miR-508-3p could be reversed by overexpression of SATB2 (n=3). (**C**, **D**) The inhibited mRNA and protein expression of SATB2 mediated by hsa_circ_0007292 knockdown was significantly reversed by overexpression of SATB2 (n=3). Data are expressed as the mean ± SD. *p < 0.05, **p < 0.01, ***p < 0.001. NS, not significant (p > 0.05).
