# Supplementary material for: Hsa-circ-0007292 promotes the osteogenic differentiation of posterior longitudinal ligament cells via regulating SATB2 by sponging miR-508-3p
Source: Aging (Albany NY). 2021 Aug 23;13(16):20192–217. doi: 10.18632/aging.203381 (PMC8436939; doi:10.18632/aging.203381)
Supplement: Supplementary Tables [file aging-13-203381-s002.pdf]

SUPPLEMENTARY TABLES

Supplementary Table 1. Details of the study subjects.

	Age	Gender	Segment	Diagnosis	Type	Cell culture	Total RNA extraction
**OPLL-1**	60	Female	C5-7	OPLL	Localized	√	
**OPLL-2**	52	Male	C4-6	OPLL	Localized	√	
**OPLL-3**	59	Male	C4-6	OPLL	Localized		√
**OPLL-4**	37	Male	C5-7	OPLL	Localized		√
**OPLL-5**	41	Female	C4-6	OPLL	Mixed		√
**OPLL-6**	54	Female	C5-7	OPLL	Segmental		√
**OPLL-7**	60	Male	C4-6	OPLL	Mixed	√	
**OPLL-8**	43	Female	C4-6	OPLL	Segmental		√
**OPLL-9**	54	Male	C4-6	OPLL	Localized	√	
**OPLL-10**	53	Female	C5-7	OPLL	Localized		√
**OPLL-11**	74	Female	C5-7	OPLL	Mixed		√
**OPLL-12**	65	Male	C5-7	OPLL	Continuous	√	
**OPLL-13**	61	Female	C5-7	OPLL	Localized		√
**OPLL-14**	46	Female	C4-6	OPLL	Localized		√
**OPLL-15**	48	Male	C4-6	OPLL	Mixed	√	
**OPLL-16**	31	Male	C4-6	OPLL	Localized	√	
**OPLL-17**	56	Female	C4-6	OPLL	Segmental		√
**OPLL-18**	57	Female	C5-7	OPLL	Segmental	√	
**non-OPLL-1**	47	Female	C4-6	CDH		√	
**non-OPLL-2**	50	Female	C4-6	CDH		√	
**non-OPLL-3**	55	Male	C5-7	CDH			√
**non-OPLL-4**	45	Female	C5-7	CDH			√
**non-OPLL-5**	50	Female	C5-7	CDH			√
**non-OPLL-6**	65	Male	C3-5	CDH		√	
**non-OPLL-7**	64	Female	C5-7	CDH			√
**non-OPLL-8**	68	Female	C5-7	CDH		√	
**non-OPLL-9**	60	Male	C4-6	CDH		√	
**non-OPLL-10**	54	Male	C5-7	CDH		√	
**non-OPLL-11**	50	Male	C3-5	CDH		√	
**non-OPLL-12**	53	Male	C4-6	CDH			√
**non-OPLL-13**	50	Female	C5-7	CDH			√
**non-OPLL-14**	40	Male	C3-5	CDH			√
**non-OPLL-15**	51	Female	C4-6	CDH			√
**non-OPLL-16**	56	Female	C4-6	CDH			√
**non-OPLL-17**	45	Male	C3-5	CDH			√
**non-OPLL-18**	62	Female	C5-7	CDH		√	

Annotation

OPLL, ossification of posterior longitudinal ligament; CDH, cervical disc herniation OPLL-10, OPLL-11, OPLL-14, non-OPLL-7, non-OPLL-15, and non-OPLL-16 were selected to perform the Microarray analysis.

Supplementary Table 2. RNA quality of the 6 tissue samples for microarray.

Sample	OD260/280	OD260/230	Concentration (ng/ul)	Volume (ul)	Quantity (ng)	QC Purity Pass or Fail
OPLL-10	1.86	2.00	130.88	30	3926.40	Pass
OPLL-11	1.89	2.06	172.90	30	5187.00	Pass
OPLL-14	2.08	1.98	997.35	20	19947.00	Pass
non-OPLL-7	1.85	2.08	176.00	30	5280.00	Pass
non-OPLL-15	1.82	1.84	101.64	30	3049.20	Pass
non-OPLL-16	2.02	1.92	338.51	15	5077.65	Pass

Supplementary Table 3. Primers used in this study.

**hsa_circ_0001588**	**F**	TGGTCTCATCTACGAGGAGACT
	**R**	CAACACACCAACGAAAATAGC
**hsa_circ_0000514**	**F**	AGCTTGGAACAGACTCACGG
	**R**	TCTCCTGCCCAGTCTGACCT
**hsa_circ_0008702**	**F**	GCAACTCTCTCTCAGATCACAA
	**R**	CCAATACATGTAAATTTGTCTGG
**hsa_circ_0007292**	**F**	ATGCTTCTGAGATGATTGACAA
	**R**	CTTTAGTCTCCTGGTGAAGAGC
**hsa_circ_0018905**	**F**	CTGTATTCTTGCTTCGCTGCTT
	**R**	CTCCTCCGCTGCCTTTCC
**hsa_circ_0003302**	**F**	CTTAATGGCAGCGTTGTTACTG
	**R**	CATCTTTCTTTCTTAAAATGCTGAA
**hsa_circ_0028540**	**F**	GAAGCCAGCGAGGATGCC
	**R**	GAAGCCAGCGAGGATGCC
**hsa_circ_0048467**	**F**	ACATGGAAGGAGGCATCTCGT
	**R**	GCTTTGCTCTGCATCGCTG
**hsa_circ_0052867**	**F**	GCAGCTCTTTCAGAGGACTAAT
	**R**	TTTCATCCACAGATCAAACCTATA
**hsa_circ_0070040**	**F**	TTGGGAACTGGACTGGGATT
	**R**	TGTTAGTTGGGGCAGAAAAGC
**hsa_circ_0000463**	**F**	TCCCAGGTCAGGCACTCC
	**R**	TGCATTCAGGAAGTCACAGTC
**hsa_circ_0002131**	**F**	ATGTTTTGGCTTTGGGGCTA
	**R**	TGGATGGAGGATGAGGATGGTA
**hsa_circ_0040994**	**F**	CTTTGCGATTCAACAAGTCAGG
	**R**	AGGCGGAGGAGGATCATTTG
**hsa_circ_0004069**	**F**	GAGCGTGCCTAGCTTCTTCTG
	**R**	GAGCGCACCAAAGACAAACA
ATP5C1	**F**	GGCAGCAAAATATGCCCGAG
	**R**	TTTTGCAGCACGTCAGCATC
**β-actin**	**F**	ATGTGGCCGAGGACTTTGATTGC
	**R**	TGTGTGGACTTGGGAGAGGACTG
**U6**	**F**	CGCTTCGGCAGCACATATAC
	**R**	AAATATGGAACGCTTCACGA

**COL1**	**F**	AAAGATGGACTCAACGGTCTC
	**R**	CATCGTGAGCCTTCTCTTGAG
**Runx2**	**F**	AGGCAGTTCCCAAGCATTTCATCC
	**R**	TGGCAGGTAGGTGTGGTAGTGAG
**OPN**	**F**	AGCAGGAGGAGGCAGAGCAC
	**R**	GTCGGCGTTTGGCTGAGAAGG
**OCN**	**F**	CTACCTGTATCAATGGCTGGG
	**R**	GGATTGAGCTCACACACCT
**miR-508-3p**	**F**	CCGCGCGTGATTGTAGCCTTTTGGAG
**miR-1179**	**F**	GCCGCGCGAAGCATTCTTTCATTGGT
**miR-485-3p**	**F**	GGCGTCATACACGGCTCTCCTCTCT
**miR-515-5p**	**F**	GCGCGCGTTCTCCAAAAGAAAGCACT
**SATB2**	**F**	GTACCATCCGTCGCTTCCTGAAC
	**R**	CGCTGTGGTGATGCCTTGACTC

Supplementary Table 4. Antibodies used in this study for western blot.

Primary or secondary	Target	Species	Concentration	Obtained from
Primary antibody	COL1	Rabbit	1:2000	AF1840, Beyotime, China
Primary antibody	Runx2	Rabbit	1:2000	ab236639, Abcam, UK
Primary antibody	OPN	Rabbit	1:2000	AF7662, Beyotime, China
Primary antibody	OCN	Rabbit	1:2000	AF6297, Beyotime, China
Primary antibody	SATB2	Rabbit	1:2000	ab92446, Abcam, UK
Primary antibody	β-actin	Mouse	1:2000	TA-09, ZSGB-BIO, China
Secondary antibody	anti-mouse-IgG	Goat	1:10000	ZB-2305, ZSGB-BIO, China
Secondary antibody	anti-rabbit IgG	Goat	1:10000	ZB-5301, ZSGB-BIO, China
